# Data-driven exploratory method investigation on the effect of dyslexia education at brain connectivity in Turkish children: a preliminary study

**DOI:** 10.1007/s00429-024-02820-5

**Published:** 2024-07-13

**Authors:** Şerife Gengeç Benli, Semra İçer, Esra Demirci, Zehra Filiz Karaman, Zeynep Ak, İrem Acer, Gizem Rüveyda Sağır, Ebru Aker, Büşra Sertkaya

**Affiliations:** 1 Department of Biomedical Engineering, Engineering Faculty, Erciyes University, Kayseri, Turkey; 2 Department of Child and Adolescent Psychiatry, Faculty of Medicine, Erciyes University, Kayseri, Turkey; 3 Department of Pediatric Radiology, Faculty of Medicine, Erciyes University, Kayseri, Turkey; 4 Department of Biomedical Engineering, Graduate School of Natural and Applied Sciences, Erciyes University, Kayseri, Turkey

**Keywords:** Dyslexia, Special education, Functional connectivity, Brain networks

## Abstract

Dyslexia is a specific learning disability that is neurobiological in origin and is characterized by reading and/or spelling problems affecting the development of language-related skills. The aim of this study is to reveal functional markers based on dyslexia by examining the functions of brain regions in resting state and reading tasks and to analyze the effects of special education given during the treatment process of dyslexia. A total of 43 children, aged between 7 and 12, whose native language was Turkish, participated in the study in three groups including those diagnosed with dyslexia for the first time, those receiving special education for dyslexia, and healthy children. Independent component analysis method was employed to analyze functional connectivity variations among three groups both at rest and during the continuous reading task. A whole-brain scanning during task fulfillment and resting states revealed that there were significant differences in the regions including lateral visual, default mode, left frontoparietal, ventral attention, orbitofrontal and lateral motor network. Our results revealed the necessity of adding motor coordination exercises to the training of dyslexic participants and showed that training led to functional connectivity in some brain regions similar to the healthy group. Additionally, our findings confirmed that impulsivity is associated with motor coordination and visuality, and that the dyslexic group has weaknesses in brain connectivity related to these conditions. According to our preliminary results, the differences obtained between children with dyslexia, group of dyslexia with special education and healthy children has revealed the effect of education on brain functions as well as enabling a comprehensive examination of dyslexia.

## Introduction

Dyslexia is a specific learning disability that is neurobiological in origin and is characterized by persistent reading and/or spelling problems affecting the development of language-related skills (Miciak and Fletcher 2020; Lyon et al. 2003). This disorder is known to affect approximately 5–17% of children and continue into adulthood (Rüsseler et al. 2018; Shaywitz 1998). In our country, it is stated that approximately 10% of school-age children have dyslexia (Çeliktürk Sezgin and Akyol 2015). While the diagnosis of dyslexia is usually made as a result of the child not being able to learn to read at the desired level in the years when a child typically starts learning to read (8–9 years of age), the age of diagnosis may be earlier or later due to some environmental (Theodoridou et al. 2021) and familial factors (Centanni et al. 2019). Dyslexia is mostly diagnosed at school ages (Yang et al. 2022), and this disorder negatively affects the child’s academic success and self-confidence (Kana et al. 2023; Wajuihian 2011; Wajuihian and Naidoo 2011). Early diagnosis of dyslexia is quite crucial as it will directly affect children’s personal development, academic success, and social life skills (Soğanci and Kulesza 2023; Huang et al. 2020; Nevill and Forsey 2023). Therefore the current study focuses on primary school children aged between 7 and 12 years old.

In recent years, various neuroimaging methods have gained remarkable importance in the diagnosis of neuropsychiatric diseases such as dyslexia (Cainelli et al. 2023; Gallego-Molina et al. 2023). Among neuroimaging methods, functional magnetic resonance imaging (fMRI) has become an extremely important tool thanks to its power in revealing the functional structure of the brain (Prasad et al. 2020). Functional MR imaging can be used to investigate the functional structure of the brain at rest without giving any stimulus, or it can be planned to show the functional structure of the brain during the designed process by giving people visual, auditory or a different stimulus/task. fMRI image analysis methods are focused on exploring functional changes in the brain, which exhibit different superior aspects, primarily emphasizing hypothesis-driven or exploratory approaches. Seed-based analysis, which examines the functional connectivity of any selected region in the brain with the whole brain or another region (Icer et al. 2018; S. İçer et al. 2023), and independent component analysis (ICA), which probes into the independent neurodynamic network structure of the whole brain with an exploratory approach as performed in this study, are among the main methods employed in this field (Icer et al. 2019).

Functional MRI studies performed specifically for dyslexia are mostly aimed at understanding the functional changes the brain shows during a stimulus or task given to individuals with dyslexia. In studies conducting reading analyzes on individuals diagnosed with dyslexia, different task configurations have been designed in the literature, resulting in notable decreases and increases in functional activation in certain brain regions (Li and H.-Y. 2022; Martin et al. 2016; Devoto et al. 2022). Studies aimed at analyzing and understanding the functional connectivity of the dyslexic brain during rest are conducted less frequently than task-based studies. (Seitzman et al. 2019; Schurz et al. 2015; Farris et al. 2011; Koyama et al. 2013; Ye et al. 2014). On the other hand, studies on resting state and task-based research examining both the resting structure of the dyslexic brain and the functional changes during dyslexia-specific neurocognitive tasks are very limited (Turker et al. 2023; Gosse et al. 2022). Ye et al. (2014) applied ICA analysis to examine dynamically modulated functional networks in the processing of incongruent and congruent words in 20 native German-speaking participants. ICA analysis has revealed that in several brain regions, such as the supplementary motor area, were modulated by the incongruous endings of sentences (Ye et al. 2014). Rüsseler et al. (2018) used independent component analysis to identify brain networks involved in the perception of audiovisual speech in a group of adult readers with dyslexia (n = 13) and a group of fluent readers (n = 13). The findings identified several components, including the fusiform gyrus and occipital gyrus, are modulated differently in fluent readers and readers with dyslexia (Rüsseler et al. 2018). Greeley et al. (2021) made an intergroup comparison with a group of readers with reading difficulties (n = 42) and a group of adolescents and children with typical reading abilities (n = 19) using rapid ICA and seed analysis. The results of ICA indicated that the group with reading difficulties exhibited alterations in sensorimotor, salience, and cerebellar networks (Greeley et al. 2021). Mohammadi et al. 2020 performed the ICA approach in adult groups consisting of 20 illiterate and 20 normal readers using resting state fMRI. In addition, literacy training was given to the illiterate group for 7 months and then the groups were evaluated with seed-based correlation analysis. Inter-group analysis revealed changes in connectivity in the left fronto-parietal network, basal ganglia network, and visual network, both before and after training (Mohammadi et al. 2020). In addition to these adult studies, the fact that dyslexia is first noticed in childhood makes it very important to investigate the functional structure and neurobiological basis of dyslexia in childhood.

Although it is not a routine method applied in the psychiatric treatment processes of individuals with dyslexia, special reading education programs are generally applied to children with dyslexia along with their school education. As a result of a comprehensive literature review conducted in this direction, the participant groups, the age range of the participants, the language used in reading tasks, the status of receiving training for dyslexia, and the rest/task studies conducted on the brain networks of interest have given in detail in Table 1.

**Table 1:**
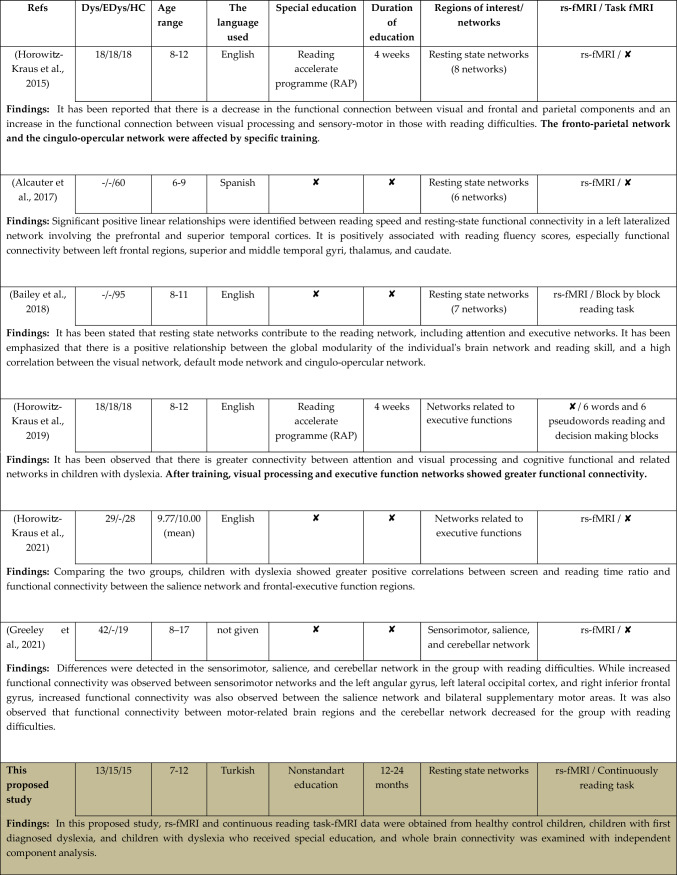
Studies performing ICA in dyslexia

As compared to recent studies in the literature detailed in the Table 1, superior aspects and unique contributions of this study can be summarized as follows.

In this study, both rs-fMRI data and reading task-fMRI data, specifically designed for our study in which the participants performed continuous reading, were obtained from participants belonging to three groups. With ICA analysis, which is an exploratory method, all brain networks and all functional connections in these networks were examined. In the literature, there are a limited number of ICA studies focusing on dyslexic children within a specific age range. In our study, the participants include children with dyslexia diagnosed for the first time (Dys), dyslexic children who received special education (EDys), and healthy control group children (HC), between the ages of 7 and 12, and ICA analysis was conducted to their fMRI data. This research is the first to employ a continuous reading task focusing on dyslexia with participants who are native Turkish speakers, by making them read a Turkish text selected according to their grade levels. Participants were asked to continuously read a text selected according to grade levels from a specific learning difficulties battery, which would be at eye level and comfortable for them to read, and MRI scans were performed. The implementation of a continuous reading task related to dyslexia, the participants' native language being Turkish, and the reading text being a Turkish text chosen according to grade level are among some of the unique aspects of the study. Participants were asked to continuously read a text from a specific learning difficulties battery, which would be at eye level and comfortable for them to read, and MRI scans were performed. Unlike few studies in the literature focusing on special education conducted at different periods of time, the inclusion of individuals who have been trained for a period of 12–24 months is expected to create a difference in functional connectivity in the dyslexia group who received special education, which forms another unique aspect of the study.

In the light of relavant literature, the aim of this study is to reveal functional markers based on dyslexia by examining the functions of brain regions during resting state and continuous reading tasks and to elaborate on the effects of special education given during the treatment process of dyslexia. In the second part of the study, information about our original data set is given and the methods used in rs-fMRI/task-fMRI analysis are detailed. In the third section, the results obtained from these methods are evaluated and compared with relevant literature findings.

## Materials and methods

The current section of the study analyzing the effects of dyslexia and special education involves the following steps: selection of participants, structural and functional data acquisition, experimental design, data preprocess and postprocess.

### Participants

A total of 43 children, whose native language is Turkish, were included in this study, comprising 13 children diagnosed with dyslexia for the first time, 15 children who received special education for dyslexia, and 15 healthy control children.

All dyslexic children were diagnosed by an experienced child and adolescent psychiatrist based on the criteria of the Diagnostic and Statistical Manual of Mental Disorders, 5th edition (DSM-5) (American Psychiatric Association.^2013^) and also the specific learning disability (SLD) battery, which comprises subtests that assess literacy and basic arithmetic skills, and tests that assess disorders or problems in visual perception, ranking and sequencing skills, the hand-eye–ear test of the head, lateralization, and fine motor skills (Turgut Turan et al. 2016), was also performed to children with dyslexia. Children with dyslexia who have any central nervous system diseases such as epilepsy, cerebral palsy, developmental delay, and who have any other psychiatric disorders were not included in the study. Children with hearing and vision problems were also excluded.

*Children diagnosed with dyslexia for the first time (Dys)*: 13 children, who are newly diagnosed with dyslexia according to DSM-V (Nevill and Forsey 2023) criteria, who have no additional psychiatric diagnosis, and who had no special education on dyslexia, aged between “7–12”, right-hand dominant, and who had an IQ level of 80 and above, were included in the study. In addition, apart from the 13 children included in the study, two children who underwent MRI were not included in the study due to the high head movement observed as a result of the pre-processing, one child could not be included because the functional MRI scan performed during the reading could not be completed successfully, and one child was not included in the study because a pathological mass was detected in the brain.

*Children with dyslexia who received special education (EDys)*: 15 children, who were diagnosed according to DSM-V criteria, with no additional psychiatric diagnosis, aged between 7 and 12, right-hand dominant, with an IQ level of 80 and above, and who received dyslexia education between 12 and 24 months, were included in the study. An individual education program is implemented for each child (https://orgm.meb.gov.tr/meb_iys_dosyalar/2021_05/21130110_Ogrenme_Guclugu.pdf, 2024).

*Healthy Control Group (HC)*: 16 children, who applied to the child psychiatry outpatient clinics of Erciyes University (ERÜ), Faculty of Medicine, between the ages of “7–12”, right-hand dominant, with an IQ level of 80 and above, and without any psychiatric disorders, were determined for the control group. Afterwards, MRI scans were performed on these children and while 15 children were included in the study, one child was excluded from the study due to high head movement observed during the preprocessing. Demographic information and varios performance values of the participants are given in Table 2.

**Table 2:** Demographic informations and performance values

	Dys (n = 13)	EDys (n = 15)	HC (n = 15)	p-values
Gender (F/M)	3/10	6/9	10/5	
Age (mean ± SD)	8.92 ± 1.7	9.4 ± 1.5	10.06 ± 1.53	0.168
Age at diagnosis (mean ± SD)	8.46 ± 1.56	7.92 ± 1.32	–	
Duration of receiving special education (month) (mean ± SD)	–	18 ± 7.52	–	
Number of words read per minute (mean ± SD)	28.69 ± 13.81	36.46 ± 21.04**	85.66 ± 19.8*	0.001
WISC_4 (mean ± SD)	84.69 ± 6.66	92.46 ± 16.34	96.6 ± 8.32*	0.042

ANOVA test; post hoc Tukey HSD

^*^Higher than Dys, **Lower than HC

This study has been approved by the Erciyes University Clinical Research Ethics Committee (Decision No: 2022/504). Written informed consent was obtained from both children and their parents.

### Data acquisition

The functional and structural MR images of each participant were acquired using a Siemens Magnetom Aera 1.5 Tesla MRI scanner with a 20-channel head coil at Erciyes University Mustafa Eraslan and Fevzi Mercan Children’s Hospital, Department of Pediatric Radiology (Icer et al. 2018).

### Structural MRI data acquisition

Structural MR data were collected with T1-weighted structural MPRAGE (magnetization-prepared rapid gradient-echo). The implemented scan parameters were sagittal orientation, echo time (TE) = 2.670 ms, repetition time (TR) = 1900 ms, 256 × 256 matrix, isotropic resolution = 1.3 mm, flip angle = 15°, and total scan time = 4 min 18 s for 192 slices, respectively. These structural MRI images were used to coregistrate the functional images to the participants’ brain anatomy during the pre-processing stage (Icer et al. 2019).

### Functional MRI data acquisition

Functional MR data were collected in an oblique plane (parallel to the anterior commissure–posterior commissure) using a T2*-weighted echo-planar imaging sequence with the following imaging parameters: TR = 2800 ms, TE = 53 ms, flip angle = 90°, field of view = 192 mm, 25 slices covering the whole brain, slice thickness = 5 mm, in-plane resolution = 2 × 2 mm. Two separate screenings were carried out for each participant as resting state and reading tasks, and each scan lasted 6 min and 14 s, and a total of 130 volumes of images were obtained at each scanning.

**Fig. 1:**
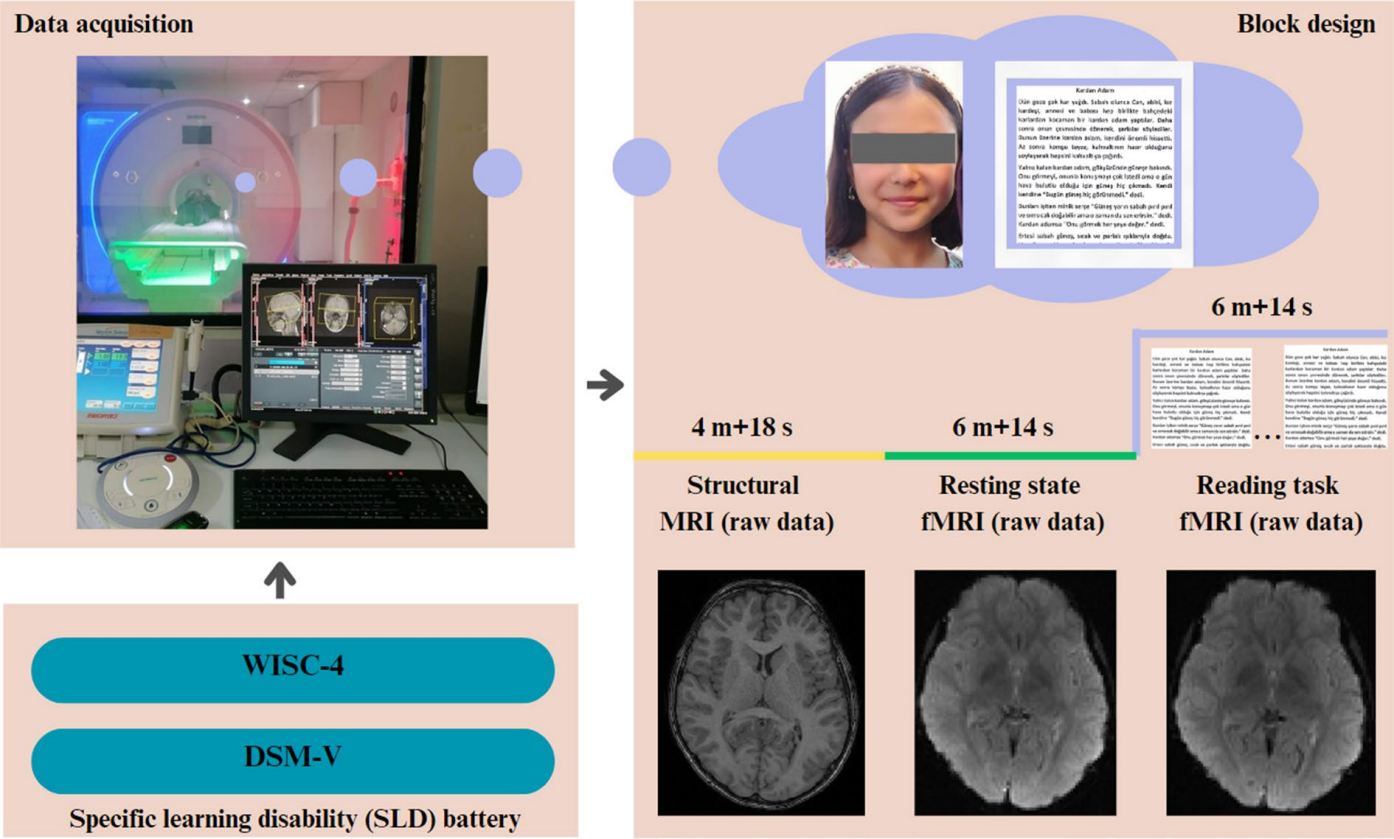
Flowchart of the whole data acquisition process

During a resting-state fMRI scan, paticipants were instructed to keep their eyes open and rest during the scan, keeping them relaxed, still, and thinking to a minimum. Under these conditions, brain activities were scanned. After the completion of the resting-state fMRI scan, a specific text determined according to the child’s grade level based on the specific learning difficulty battery (Karakaş et al. 2017) was placed in the upper inner region of the MRI device at the child’s eye level. Texts in different font styles and font sizes were arranged for children to read. These texts were written in children’s native language (Turkish). Participants were primary school 2, 3 and 4th grade students. Therefore, three different texts were separately prepared for students at different grades. All texts were selected from textbooks issued by the Ministry of National Education, Republic of Turkey. Relevant texts were selected in a way that the child could comfortably read based on their academic level, and the task given to the child was to silently read through the text and then start reading it again from the beginning after completing it. This reading action was continued throughout the scanning period, and brain activity was examined. Unlike the stop-and-go tasks commonly seen in the literature, a continuous task condition was implemented. Figure 1 illustrates the methodological framework of the study.

### Data preprocessing

Pre-processing steps for functional MR images are practically carried out with auxiliary software. In this study, the data preprocessing process was carried out with the FMRIB Software Library (FSL 6.0.4) (https://fsl.fmrib.ox.ac.uk/fsl/fslwiki). The same preprocessing steps were performed for the resting state and the continuous reading task. Relavant preprocessing steps used in the study are as follows; brain extraction, slice timing correction, motion correction, spatial smoothing, ICA AROMA, temporal filtering and linear domain registration.

Initially, non-brain structures were extracted from anatomical and functional images using FSL FMRIB's brain extraction tool (BET) (Smith 2002). Next, slice timing correction was performed by shifting all slices to be aligned as if they were acquired at the same point and time using voxel time series interpolation relative to a reference slice. Motion correction was carried out using the middle volume as the initial template and applying 6 degrees of freedom (DOF)—3 rotations and 3 translations—to each volume with FLIRT optimization method to remove motion artifacts (Jenkinson et al. 2002). Children with estimated maximum absolute head motion > 2 (mm or °) or mean motion > 0.5 (mm or °) were excluded from the study to minimize the effects of unwanted motion (Winkler et al. 2014; Sörös et al. 2019). Spatial smoothing involves obtaining the intensity value of each voxel by taking the mean of the values of neighboring voxels, thereby reducing high-frequency fluctuations between adjacent voxels (Chen and Calhoun 2018).

In the study, a low-pass Gaussian filter was applied to the images, eliminating high-frequency signals and preserving low-frequency information. The smoothing process was performed using a Gaussian kernel with FWHM = 5 mm in each fMR image volume separately. After spatial smoothing, FSL’s ICA-AROMA tool (version 0.3-beta), an automatic motion artifact removal tool based on independent component analysis, was used to detect artifacts and then remove these components from the data (Pruim et al. 2015). The ICA AROMA process was carried out after smoothing and before filtering. By means of AROMA, temporal filtering was applied to functional images to eliminate motion artifacts. Through temporal filtering, low-frequency artifacts were removed with high-pass temporal filtering using the local form of a straight line with cut-off = 100 s = 0.01 Hz, which is the recommended value for fMRI data (Smith et al. 2004). The functional data were registered to the high-resolution anatomical images using a 6-degree-of-freedom linear transformation (DOF) applied in the FLIRT linear registration tool available in FSL (Jenkinson et al. 2002).

**Fig. 2:**
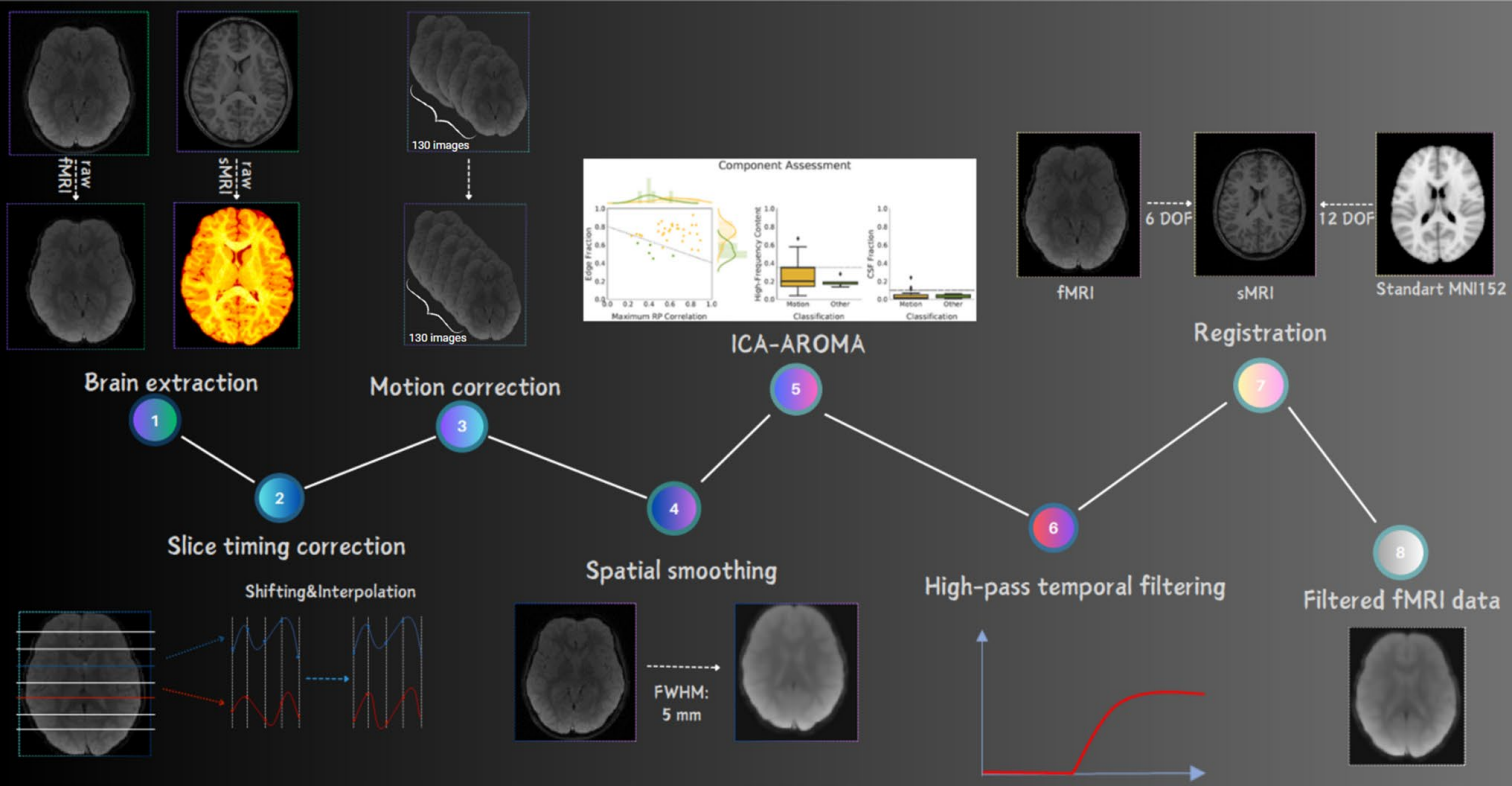
Flowchart of fMRI data preprocessing

To overcome some of the shortcomings of FLIRT, registration of structural images to the 2-mm MNI standard space template was performed using a 12-degree-of-freedom linear transformation through the nonlinear registration tool FNIRT (Andersson et al. 2008). This ensures better alignment of the structures. Finally, the low-resolution fMRI image was registered to the standard space, and the two transformations were merged. The preprocessing steps applied to anatomical and functional images are illustrated in Fig. 2.

### Functional connectivity analysis

In this study, independent component analysis (ICA) was applied to fMRI data obtained during the resting and reading states. We performed ICA analysis on group data using FSL’s MELODIC software (https://fsl.fmrib.ox.ac.uk/fsl/fslwiki/MELODIC/), which implements probabilistic ICA. The methodology used to make group inferences when performing group ICA analysis in the MELODIC tool is multi-session temporal concatenation. This method is most suitable for resting-state data as there is no common time course for subjects, unlike the task-oriented design. Temporal ICA combines single-subject data sets over time and obtains independent components on the combined data matrix (Vos et al. 2018). Since the reading task in our study was continuous, an analysis method equivalent to resting state ICA analysis was applied. In this study, the number of independent components determined for both the resting state and the reading task was 20. As a result of group ICA analysis, 20-component ICA maps were obtained (Smith et al. 2009; Biswal et al. 2010; Wang and Li 2015).

**Fig. 3:**
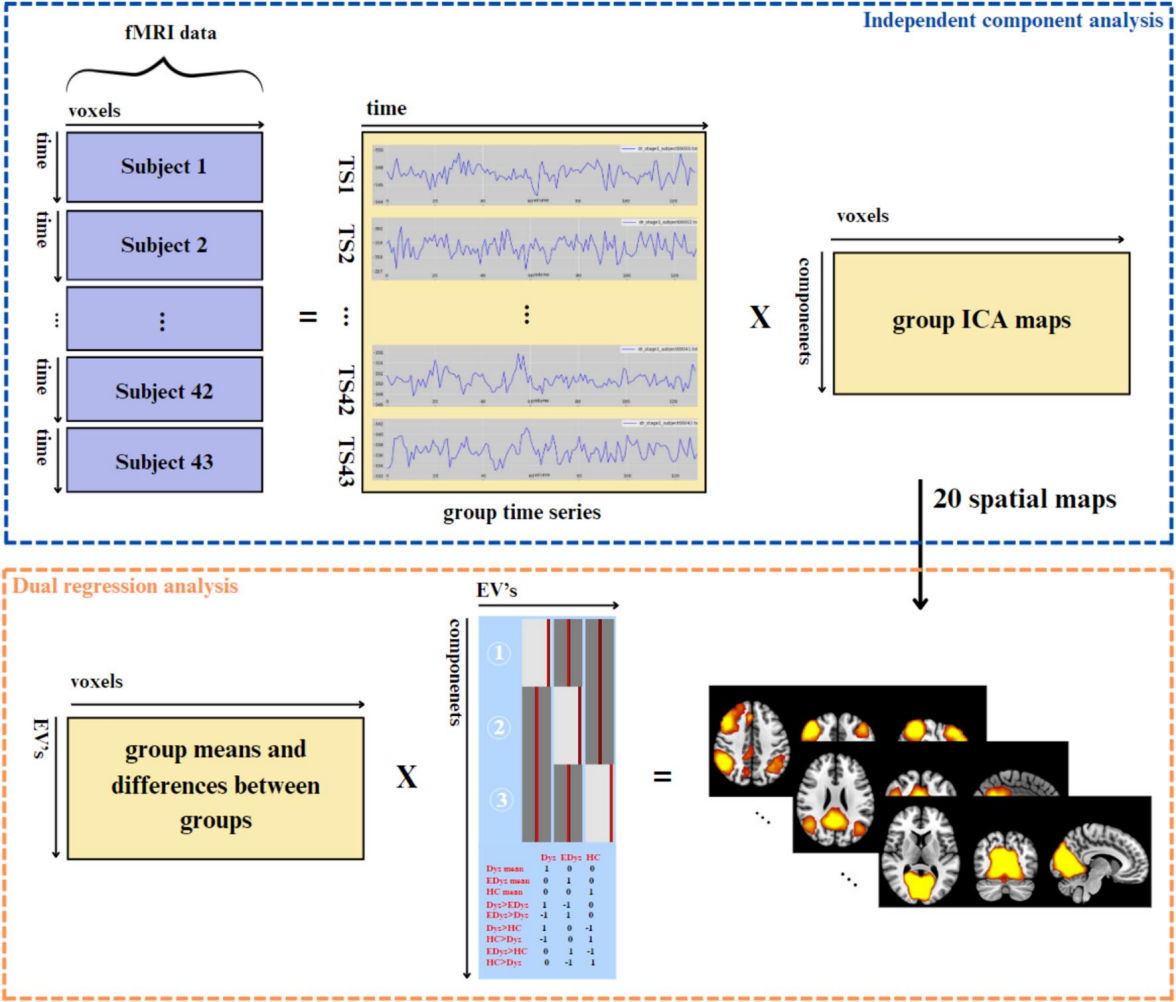
Flowchart of independent component analysis and dual regression analysis

In this study, after multi-subject group ICA analysis were completed, dual regression was performed on FSL to evaluate the functional connectivity differences between Dys, EDys and HC groups (Andersson et al. 2008). Dual regression allows the definition of a set of network maps and corresponding time series to be compared between groups associated with group ICA components within each individual’s spatial space (Vos et al. 2018). ICA maps obtained as a result of group ICA were utilized as input in dual regression, in which multivariate temporal regression was performed to evaluate individual spatial maps using time courses. Group analysis was then performed by entering participants’ individual independent component (IC) spatial maps into a generalized linear model (GLM) framework using an appropriate design matrix and corresponding contrasts. Independent component analysis and dual regression analysis are summarized in Fig. 3.

In the general linear model, three explanatory variables are defined as follows: Dys, EDys and HC groups. Then, 2 sample t-test contrasts corresponding to all possible combinations were defined to evaluate the differences between groups (6 contrasts) (https://fsl.fmrib.ox.ac.uk/fsl/fslwiki/GLM).

Binary regression with variance normalization was performed to demonstrate the activity and spatial extent of resting-state networks (RSN). Regarding statistical analysis, different component maps were collected in 4D files across participants and tested voxel-wise for statistically significant differences between groups using FSL’s randomized tool performing non-parametric permutation testing. In order to check through multiple comparisons, 5000 permutations were applied for each indicated contrast using the Threshold free cluster enhancement (TFCE) technique. Finally, family wise error (FWE) correction was performed for multiple comparisons applying TFCE using the significance threshold of p < 0.05. Regions with differences between groups were used to extract mean z values from each spatial map (FWE-corrected p < 0.05) (Winkler et al. 2014; Rytty et al. 2013).

## Results and discussion

**Fig. 4:**
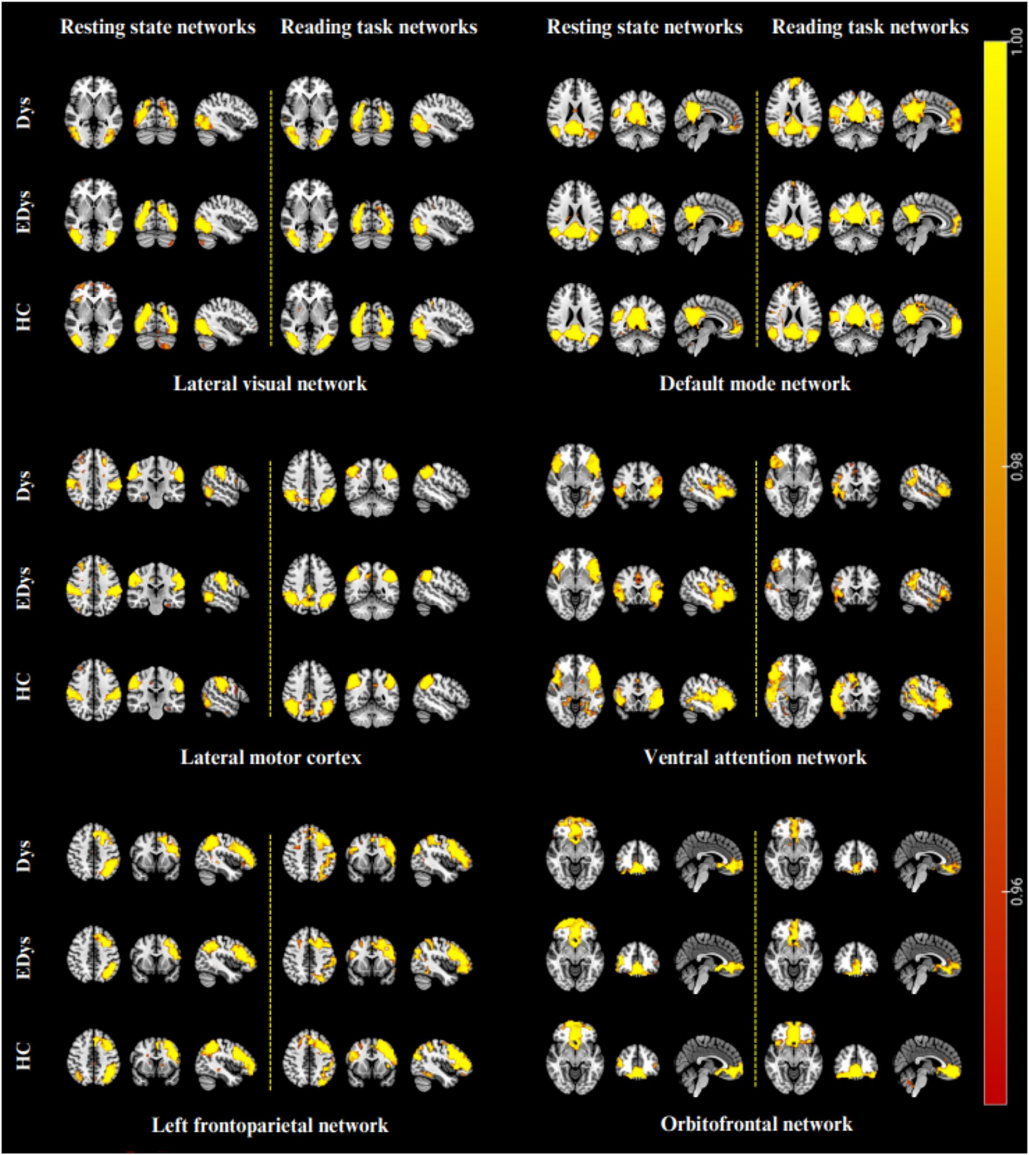
Mean networks of each group after dual regression

In this study, 20-component ICA analysis was applied to fMRI data obtained from children during both resting-state and reading task fMRI scans, and the results obtained for networks and regions specifically relevant to our study are presented. Figure 4 provides group means of brain networks corresponding to 6 independent components for reading task and resting-state conditions. Table 3 indicates voxel-wise activations in regions of interest within the relevant brain networks for both sessions for each group.

**Table 3:**
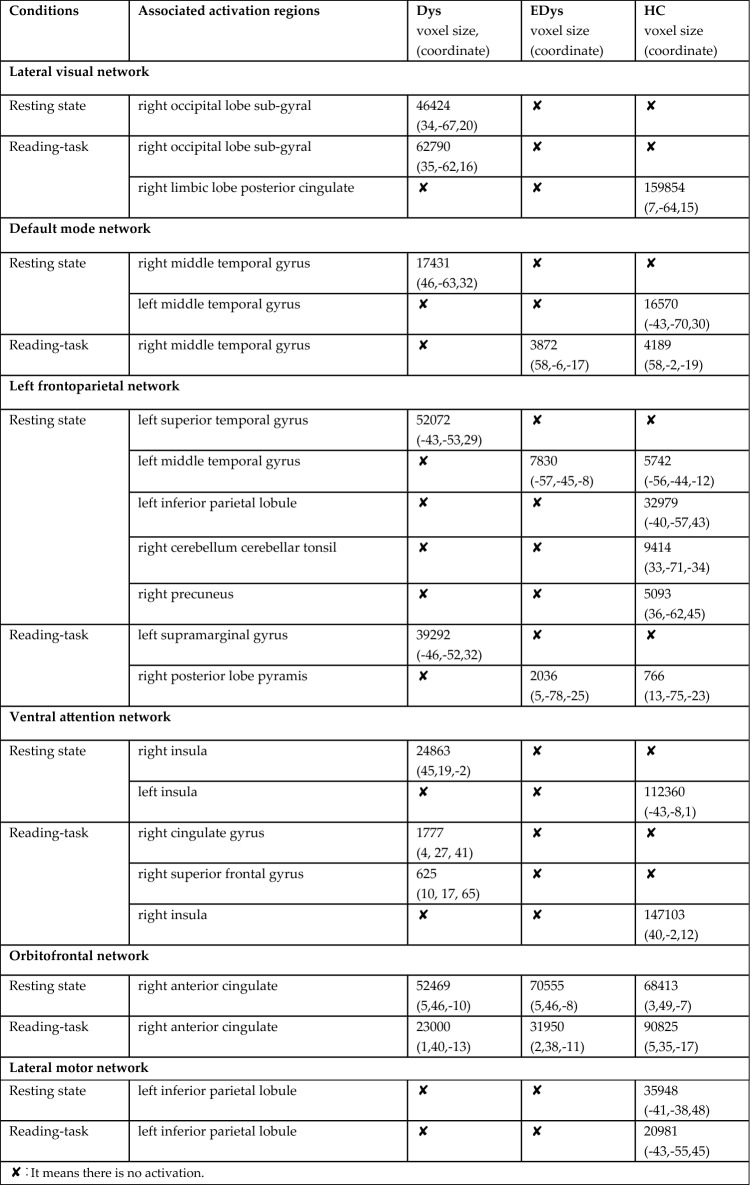
Reading task and resting state mean group networks and activation regions

**Fig. 5:**
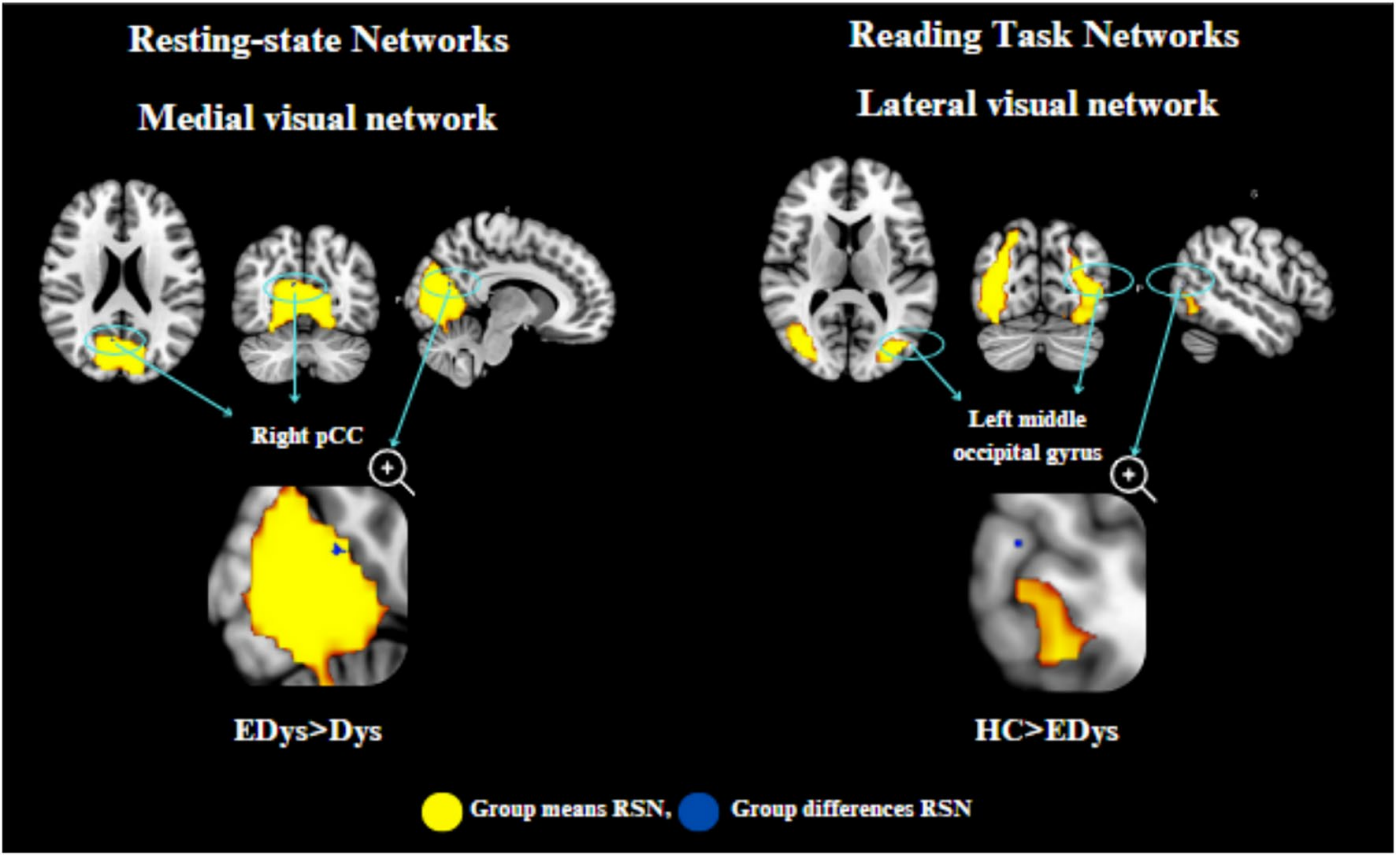
Significant connectivity differences between groups in resting state and reading task

As a result of the ICA analysis, dual regression analysis was applied to the networks formed in both the resting state and the reading task in order to see the differences between the groups. With the dual regression analysis (comparison between groups) performed for resting and reading task, the regions showing significant differences are presented numerically and visually in Table 4 and Fig. 5.

**Table 4:** Resting state and reading task dual regression analysis results

Group contrast	EDys > Dys medial visual network (rs-fMRI)	HC > EDys lateral visual network (task-fMRI)
Associated activation region	Right posterior cingulate	Left middle occipital gyrus
MNI coordinate	9, 6, 21	51, 75, 11
Voxel size	30	7
p-value	0.9648	0.9816

The findings obtained for the networks of interest within the scope of this study were evaluated by taking into account Table 3, which was obtained based on group means.

### Visual network

During the resting state, activation was observed only in the Dys group in the sub-gyral region of the right occipital lobe in the lateral visual network, while during the reading task, activation was again observed only in the Dys group in the same region. However, it is noteworthy that there is increased activation during the reading task compared to resting state. Visual processing difficulties could be attributed to the right occipital lobe sub-gyral.

According to the results of the second-level analysis, during the reading task in the lateral visual network, it was found that there was greater activation in the left middle occipital gyrus region in HC compared to EDys (HC > EDys). Again, the difference in activation in the left middle occipital gyrus observed in healthy controls during the reading task (Table 4; Fig. 5) can be considered as a finding that details such as distance and depth in these children with dyslexia, they have not yet developed as much as healthy children, despite receiving training. According to the second level analysis results, it was found that there was more activation in the right PCC region in Edys than in Dys (EDys > Dys) during rest in the medial visual network.

Considering the relationship between PCC and anxiety (Gorka et al. 2023), the increased connectivity at the right PCC observed in dyslexics who received education during rest (Table 4) can be considered a finding of the effect of special education on both anxiety and episodic memory-related (Alsulami 2019; Stoitsis et al. 2008) differences in these children. This situation may be a sign of improvement in anxiety. As seen in Gorka’s study, PCC has been reported to be associated with increased task performance of anxiety, supporting our results (Stoitsis et al. 2008). In a study featuring an audiovisual task, increased activation in the right cingulate gyrus region was observed in the *HC* group compared to the *Dys* group (Kronschnabel et al. 2014). Barquero et al. (2014) conducted a systematic review of the literature on reading intervention in children and adults with studies using fMRI and MEG imaging methods. According to the findings obtained from the meta-analysis, it was stated that participants with reading difficulties manifested increased functional activation following the reading intervention in the right posterior cingulate and left middle occipital gyrus. As a result of their studies, it was found that these regions are probably active in processes that will improve reading ability (Barquero et al. 2014).

### Default mode network

In the default mode network, the right middle temporal gyrus region was activated only in the Dys group at rest, in which activation increased in both the Dys and HC groups during reading. Considering the role of MTG in language (Briggs et al. 2021; Zhang et al. 2017), semantic memory (Xu et al. 2015), visual perception (Stein 2014), and multimodal sensory integration (Mesulam 1998), the activation of the right middle temporal gyrus (MTG) in the EDys and HC groups during the reading task can be considered a benefit of the training. When the default mode network was evaluated at rest, functional activation was observed only in Dys group in the right middle temporal gyrus region and only in HC group in the left middle temporal gyrus. However, unlike the right MTG which does not activate during rest in educated individuals, it is observed that the left MTG activates during rest in HC group. This situation can also be considered as one of the connectivity differences between healthy individuals and dyslexics.

In a study by Gosse et al., involving dyslexic children (n = 16) and healthy control (n = 16) group with a mean age of 9.3, it was emphasized that there was a decrease in functional connectivity in the left middle temporal gyrus regions during resting-state in dyslexic children. The function of the middle temporal gyrus is critical for reading as it is responsible for the retrieval of visually presented items (Gosse et al. 2022). In a study by Schurz et al., examining brain areas related to reading in dyslexic readers (n = 15) and typical readers (n = 14) aged 16–20 years, both task-based and resting-state functional connectivity analyses were conducted. They revealed a decreased functional connectivity between the left middle temporal gyrus and inferior frontal gyrus in dyslexic readers (Schurz et al. 2015). This finding was consistently obtained for two different reading tasks and resting-state conditions. However, a meta-analysis study involving Chinese dyslexic children reported hyperactivation in the right middle temporal gyrus region (Li and H.-Y. 2022).

### Frontoparietal network

In the left frontoparietal network, during reading, activation was observed only in Dys in the left supramarginal gyrus, while activation was observed in EDys and HC in the right posterior lobe pyramid. This situation may indicate that EDys approaches HC with training. However, activation in the right posterior lobe pyramidis is observed during reading in both HC and Edys, but not in Dys. During resting state, activation is observed in the left inferior parietal, right cerebellum, and right precuneus in HC, with no differences found in EDys and Dys. However, activation is seen in both Edys and HC in the left middle temporal gyrus, while only Dys lacks activation. In the left superior temporal gyrus, it is seen that there is activation only in Dys. In this context, it is seen that EDys group begins to resemble HC group in some regions where the connectivity of Dys group is very different from HC.

In another study involving 16 dyslexic children and 15 children with typical readers, aged between 8 and 16, all participants performed three tasks (phonological, picture naming and semantic (3 types) tasks) during fMRI acquisition. During the complex sentence reading task, activation in the bilateral superior temporal gyrus was observed in the dyslexia group. When the dyslexic group was compared to typical readers during the same task, functional activation was observed in the right superior temporal gyrus (Prasad et al. 2020). Additionally, in a study involving dyslexic readers (n = 13, mean age = 24) and typical reader (n = 13, mean age = 25.3) where auditory-visual stimuli were presented during a reading task, it was shown that typical readers exhibited greater superior temporal activations in scenarios with combined auditory-visual stimuli compared to auditory/visual stimuli alone. On the other hand, such an increase effect is not found in dyslexic readers. In one study, the superior temporal gyrus plays a critical role in the integration of acoustic and visual speech, thus highlighting the potential of superior temporal dysfunction to underlie weak auditory-visual integration in dyslexia (Ye et al. 2017). In another study involving auditory and visual stimuli, Rüsseler et al. designed a task-based study to examine audio-visual speech perception in dyslexic individuals, comparing them with 13 dyslexic and 13 typical readers. The study findings revealed that, when comparing consistent stimuli with inconsistent stimuli, the typical reader group showed increased activation in the bilateral superior temporal gyrus, while in the dyslexic group, this pattern was reversed (Rüsseler et al. 2018).

Unlike the foregoing studies in which audio-visual stimulation was employed, this study only incorporates the continuously text reading task. Given relavant findings in the literature, this indicates that the results in our study may vary depending on the experimental paradigm. In addition, the fact that there are differences in the bilateral superior temporal gyrus between the dyslexic group and the healthy group underpins the functional significance of this region in connection with the dyslexia disorder and the specific stimulus given.

### Ventral attention network

It is known that there are morphological differences in the insula in the dyslexia group (Black 2004**).** However, the lack of activation in the left insula in the dyslexia group in functional studies draws attention as a finding similar to our study. It is known that the insula region is associated with emotional regulation (Jang et al. 2018). Since the left insula is affected in dyslexia, the activation of the right insula, which is thought to be due to compensation, has been supported by other functional imaging studies (Paulesu et al. 1996).

In addition, it is suggested that the left insula, which is not active in dyslexics unlike healthy controls in PET scans (Paulesu et al. 1996) of both different tasks, has an important role in connecting different phonological codes. If these results are reproducible, the value of studying pathological groups to determine the functional anatomy of the normal brain system will be confirmed (Paulesu et al. 1996**).** In a task-based study conducted by Łuniewska et al., involving typical readers (n = 90) or individuals with dyslexia (n = 20), with children at familial risk for dyslexia (n = 55) and those without any familial risks (n = 35), fMRI scans were performed at the beginning of primary school and repeated 2 years later. In dyslexic children, low activation was observed in the right insula during the initial scan, while over the 2 year period, brain activation during phonological processing increased in the right insula. In typical readers, a decrease in brain activation was observed in the left hemisphere’s language areas, including the insula, after 2 years (Łuniewska et al. 2019).

In our study, in the ventral attention network, the right insula region was activated in Dys group in the resting state, while the left insula region was activated in HC group. However, it was observed that only the right insula was activated in the healthy group during reading. In addition, no activation was observed in the right cingulate gyrus and right superior frontal gyrus regions in EDys and HC groups during the reading task, while activation was observed only in Dys group.

### Orbitofrontal network

It was maintained in some studies that there is a potential relationship between the right anterior cingulate gyrus and vision within the orbitofrontal network (Shinoura et al. 2013). Also, the dorsal ACC modulates the tracking of visible targets, visual attention, and the interface between cognition and emotion, thereby affecting emotional self-control and problem-solving capacity. In a study conducted on Brazilian children, a mixed experiment related to the event was carried out using a meaningful word and pseudoword reading test. Participants were asked to choose “Yes” or “No” options when asked about the meaningfulness of the word. As a finding of the reading task in the study, it was revealed that in typical readers, there was more activation in the left anterior cingulate cortex (ACC) dorsal part compared to dyslexic readers (Buchweitz et al. 2019).

In our study, in the orbitofrontal network, activation was observed in the right anterior cingulate gyrus during both rest and reading state in all three groups. However, during reading, there was an increase in connectivity compared to resting state in the healthy group, while a decrease was observed in Dys and EDys groups. The increase in activation seen during reading in HC was not found in the Dys and EDys groups; on the contrary, there was a decrease in activation, which was thought to be related to visual processing. Considering the visual function of this region, the anterior cingulate cortex should be evaluated in addition to focusing only on the temporooccipital region regarding visual processing (Huda et al. 2020). Also, regarding the relationship of this area with impulsivity (Baker and Ireland 2007), it could be said that healthy individuals may able to control their impulsivity better than dyslexia.

### Lateral motor network

In the literature, it was reported that the connection between the inferior parietal lobule (IPL) and certain cortical areas is decreased in dyslexic readers both during tasks and at rest (Schurz et al. 2015). Additionally, dysfunction of the IPL in dyslexic children has been reported in numerous studies (Maisog et al. 2008; Richlan et al. 2009, 2011). In our study, in the lateral motor network, activation is observed in the left inferior parietal lobule of the lateral motor cortex during both rest and reading in HC group, while activation is not observed in either EDys or Dys groups. The significant differences observed between healthy and dyslexic groups in both reading and resting conditions may highlight the presence of impairments in motor coordination and phonological awareness (Martin et al. 2016; Devoto et al. 2022; Pellegrino et al. 2023) in the clinical presentation of neurodevelopmental disorders. Considering this aspect, the necessity arises to integrate existing interventions aimed at improving motor coordination into special education programs. It also draws attention to the need to review the impact of existing phonological awareness studies.

A potential limitation of functional connectivity/activation studies based on reading tasks is that different reading tasks may produce different functional connectivity patterns. As noted by Koyama et al., there is still no consensus on the “optimal” task for characterizing the neural networks underlying reading and dyslexia through reading-based functional connectivity research. It is emphasized that one possible optimal solution is to examine functional connectivity during both reading tasks and resting state conditions (Koyama et al. 2010).

## Conclusions

We can summarize main findings in our study, together with the results and comments we highlighted above, as follows.

- It has been revealed that there is a need to integrate phonological awareness and motor coordination activities into dyslexia education in accordance with the findings of lateral motor networks.

- In dyslexic groups, it has been observed that dyslexia oriented educational activities contribute to the improvement in functional connectivity of certain brain regions making them closer to that of healthy groups.

- Most brain regions that yield significant results in our study appear to be in common with the literature. Considering the current results, the functional connectivity differences in our study support the need to analyze many factors such as motor skills, phonological awareness, impulsivity and anxiety in dyslexia.

The unique aspects of this study are that all participants were asked to perform both resting and continuous reading tasks, and that it consisted of three compatible data groups, such as MRI protocols and pre-processing processes as well as demographic characteristics of participants. The study’s other distinctive aspects are that the participants read the Turkish text selected according to their grade levels and that all participants were Turkish and their mother tongue was Turkish, ensuring nationality and language compatibility. In addition, it was ensured that participants with dyslexia did not have any other psychiatric diseases that could be confused with dyslexia, and children with pure dyslexia were included in the study.

One limitation of our study is the small sample size to reach a generalizable interpretation over results. The preliminary results presented in this study can be studied in a more comprehensive way by increasing the number of data and applying more diverse functional analysis methods.

## Data Availability

This study has been carried out within the scope of the TÜBİTAK project and data sharing cannot be performed yet.

## Declarations

### Permission to reproduce material from other sources

The figures and materials used were specifically prepared for this study.
